# Evaluation of Neural Degeneration Biomarkers in the Prefrontal Cortex for Early Identification of Patients With Mild Cognitive Impairment: An fNIRS Study

**DOI:** 10.3389/fnhum.2019.00317

**Published:** 2019-09-06

**Authors:** Dalin Yang, Keum-Shik Hong, So-Hyeon Yoo, Chang-Soek Kim

**Affiliations:** ^1^School of Mechanical Engineering, Pusan National University, Busan, South Korea; ^2^Department of Cogno-Mechatronics Engineering, Pusan National University, Busan, South Korea

**Keywords:** functional near-infrared spectroscopy (fNIRS), mild cognitive impairment (MCI), linear discriminant analysis (LDA), convolutional neural network (CNN), neural degeneration

## Abstract

Mild cognitive impairment (MCI), a condition characterizing poor cognition, is associated with aging and depicts early symptoms of severe cognitive impairment, known as Alzheimer’s disease (AD). Meanwhile, early detection of MCI can prevent progression to AD. A great deal of research has been performed in the past decade on MCI detection. However, availability of biomarkers for MCI detection requires greater attention. In our study, we evaluated putative and reliable biomarkers for diagnosing MCI by performing different mental tasks (i.e., *N-*back task, Stroop task, and verbal fluency task) using functional near-infrared spectroscopy (fNIRS) signals on a group of 15 MCI patients and 9 healthy control (HC). The 15 digital biomarkers (i.e., five means, seven slopes, peak, skewness, and kurtosis) and two image biomarkers (*t*-map, correlation map) in the prefrontal cortex (PFC) (i.e., left PFC, middle PFC, and right PFC) between the MCI and HC groups were investigated by the statistical analysis, linear discriminant analysis (LDA), and convolutional neural network (CNN) individually. The results reveal that the statistical analysis using digital biomarkers (with a *p*-value < 0.05) could not distinguish the MCI patients from the HC over 60% accuracy. Therefore, the current statistical analysis needs to be improved to be used for diagnosing the MCI patients. The best accuracy with LDA was 76.67% with the *N*-back and Stroop tasks. However, the CNN classification results trained by image biomarkers showed a high accuracy. In particular, the CNN results trained via *t*-maps revealed the best accuracy (90.62%) with the *N-*back task, whereas the CNN result trained by the correlation maps was 85.58% with the *N*-back task. Also, the results illustrated that investigating the sub-regions (i.e., right, middle, left) of the PFC for detecting MCI would be better than examining the whole PFC. The *t*-map (or/and the correlation map) is conclusively recommended as an image biomarker for early detection of AD. The combination of CNN and image biomarkers can provide a reliable clinical tool for diagnosing MCI patients.

## Introduction

Alzheimer’s disease (AD) is a degenerative brain disorder of unknown etiology, a common form of dementia, which begins in middle-aged or older adults (Ieracitano et al., 2018). AD results in progressive memory loss, thinking impairment, disorientation, changes in personality and mood (Niu et al., 2013). In the final stages of AD, people lose the ability to communicate or respond to their environment. They need assistance in all their activities of daily living, and they may even lose their ability to swallow. As reported via the Alzheimer’s Association, by 2050 one new case of AD is expected to develop every 33 s resulting in nearly 1 million new cases per year (Alzheimer’s Association, 2018). In addition, in 2017, more than 16 million family members and other unpaid caregivers, a contribution valued at more than $232 billion, were devoting toward the care of Alzheimer’s patients. Such findings highlight the requirement for solutions to prevent dementia-related costs from jeopardizing the health and financial security of the families of people with Alzheimer’s related diseases.

However, there is a relative mild condition of cognitive impairment before the onset of AD, known as mild cognitive impairment (MCI), a stage at which treatment can reduce the chance for developing to AD (Yeung et al., 2016b; Fang et al., 2018; Valenzuela et al., 2018). MCI patients are divided into two categories; amnestic and non-amnestic. In the case of amnestic MCI patients, the memory is affected primarily. For the case of non-amnestic MCI, the patients have difficulty with thought process such as planning and completing complex tasks such as balancing a checkbook or making a judgment in a risky situation (Marmarelis et al., 2017). There are various methods to diagnose an MCI patient. Primarily, the diagnosis in a clinic relies on the patient’s medical history and clinical rating scores, such as clinical dementia rate or Mini-Mental State Examination (MMSE) (Li R. et al., 2018). However, it is known that the MMSE performance is influenced by education and age, and the clinical evaluation and diagnosis through MMSE requires an experienced clinician (Nguyen et al., 2008). To cope with these issues, the biomedical examination methods using brain signals have been introduced, such as the transcranial Doppler ultrasonography (Keage et al., 2012), functional near-infrared spectroscopy (fNIRS) (Vermeij et al., 2017), functional magnetic resonance imaging (fMRI) (Khazaee et al., 2017; Katzorke et al., 2018), and positron emission tomography (Beishon et al., 2017). fNIRS is a relatively new optical imaging technology that uses light in the near infrared range to monitor the hemodynamic responses non-invasively: A neural firing increases blood flow in the neighboring capillary network, and fNIRS measures the concentration changes of the oxyhemoglobin (ΔHbO) and deoxyhemoglobin (ΔHbR) in the cerebral cortex (Boas et al., 2014; Hong et al., 2014; Zafar and Hong, 2018). fNIRS is known for its portability, non-invasiveness, low cost, and high temporal resolution (compared with fMRI) (Ferrari and Quaresima, 2012; Hong and Santosa, 2016; Pinti et al., 2018). Recently, the possibility of improving the spatial and temporal resolutions using a bundled-optodes configuration and the initial dip was demonstrated in the process of brain-computer-interfaces (Nguyen and Hong, 2016; Zafar and Hong, 2017; Hong and Zafar, 2018). Therefore, fNIRS has distinct advantages over other modalities (Ghafoor et al., 2017; Yap et al., 2017).

The difficulty in diagnosing the causes of diseases has a severe frustration on patients if they do not receive an appropriate care in a timely manner. Therefore, robust and sensitive biomarkers for a prompt monitoring of cognitive or biological changes between healthy elderly and MCI patients is required (Nestor et al., 2004). A number of studies have examined the feasibility of using fNIRS to diagnose MCI and other types of dementia using different biomarkers (Niu et al., 2013; Katzorke et al., 2017, 2018; Perpetuini et al., 2017; Vermeij et al., 2017; Yap et al., 2017; Halliday et al., 2018; Stuart et al., 2018). Appropriate biomarkers may provide a reliable diagnosis for patients with MCI before the onset of AD. Table 1 lists the existing biomarkers examined in the previous fNIRS studies.

**TABLE 1:** List of fNIRS biomarkers, mental tasks, and brain regions used in various studies.

**No.**	**Author (Year)**	**Biomarkers**	**Mental Task**	**Brain Region**
1	Li R. et al., 2018	Mean, Slope of ΔHbO	Digital verbal span	Frontal and bilateral parietal
2	Jung et al., 2018	Clinical assessment	Working memory	Prefrontal
3	Katzorke et al., 2018	Mean ΔHbO	Verbal fluency	Prefrontal
4	Li X. et al., 2018	Multi-scale entropy	Resting state	All scalp
5	Perpetuini et al., 2017	Entropy	Working memory	Prefrontal
6	Yap et al., 2017	No. active chs., Mean, Slope, Peak time	Verbal fluency	Prefrontal
7	Katzorke et al., 2017	Mean ΔHbO	Verbal fluency	Inferior frontal
8	Vermeij et al., 2017	Mean ΔHbO, Mean ΔHbR	Working memory	Prefrontal
9	Marmarelis et al., 2017	Cerebral autoregulation	Resting state	Prefrontal
10	Uemura et al., 2016	Mean ΔHbO	Memory retrieval	Prefrontal
11	Yeung et al., 2016b	Mean ΔHbO	Working memory	Frontal and temporal
12	Yeung et al., 2016a	Mean ΔHbO of active channels	Category fluency	Prefrontal
13	Haworth et al., 2016	Reaction time	Trail making	None
14	Heinzel et al., 2013	Mean ΔHbO	Verbal fluency	Frontal and bilateral parietal
15	Doi et al., 2015	Mean ΔHbO	Dual-task walking	Prefrontal
16	Niu et al., 2013	Mean and *t*-map of ΔHbO	Working memory	Frontal and Temporal
17	Arai et al., 2006	Mean ΔHbO	Verbal fluency	Frontal and bilateral parietal

**FIGURE 1:**
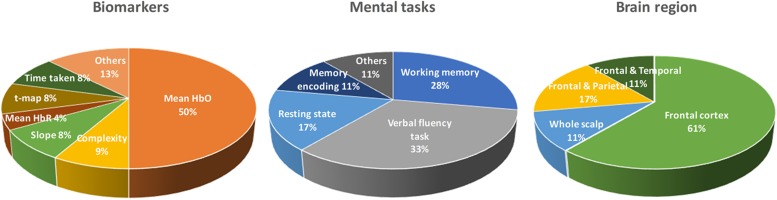
Summary of widely used biomarkers, categories of mental tasks, and brain regions used in various fNIRS studies for diagnosing of MCI patients (the total number of fNIRS articles was 17).

As shown in Table 1, there are a number of studies that have applied different mental tasks in various brain regions to assess meaningful biomarkers. Li R. et al. (2018) asked the subjects to perform a cognitive task (digit verbal span task) while brain signals were measured from the frontal and bilateral lobes. The results showed that the mean value of ΔHbO (i.e., MHbO) and the slope of ΔHbO (i.e., SHbO) were higher in healthy control (HC) than the MCI group during the time window of 3–12 s. Katzorke et al. (2018) also evaluated the biomarker of MHbO and the mean value of ΔHbR (i.e., MHbR) when the subjects performed a verbal fluency task (VFT). A slight decrease in the hemodynamic response was observed in the inferior frontotemporal cortex in the MCI group. Some of the studies have employed a quantitative analysis of multiscale entropy: The results demonstrated that the resting-state brain signal complexity was decreased in the MCI group (Perpetuini et al., 2017; Li X. et al., 2018). Yap et al. (2017) employed biomarkers such as active channels, MHbO, time response of ΔHbO to reach the peak, and SHbO for detecting a patient with MCI or AD. The results illustrated that MCI exhibited a greater mean activation (than AD and HC) for both the right and left prefrontal cortex (PFC) when the subjects performed VFT (see Figure 5B). The results using the time to reach the peak and SHbO presented a meaningful difference between the left and right PFC (see Figures 5C,D). The biomarker of using activated channels did not show a significant difference among various brain regions. The authors also claimed that the difference in the hemoglobin responses in the left and right PFC was caused by neural compensation, and that the capacity for such neural compensation was inversely proportional to the severity of neurodegeneration (Price and Friston, 2002). Figure 1 summarizes the existing biomarkers, categories of mental tasks, and brain regions that have been used in the fNIRS studies for diagnosing the patients with MCI.

Even there exist a number of biomarkers in the fNIRS area as in Table 1. Most of the studies prefer to conduct the statistical analysis for seeking the group difference between the MCI and HC. However, the high standard deviations (SD) illustrate that the method of using statistical analysis is not useful in establishing a confident diagnosis of individual patients for clinical purposes (Labaer, 2005). To the best of authors’ knowledge, there is no result on the evaluation the existing biomarkers, brain regions, and time durations. Cotelli et al. (2008) and Park and Reuter-Lorenz (2009) suggested the right PFC as one of the functional compensatory regions in cognitively impaired individuals. Additionally, the selection of a proper biomarker will directly influence the results on classification and diagnosis of the disease. Therefore, the evaluation of the digital biomarkers, brain regions, and time intervals in obtaining biomarkers is necessary, and it would become a reference for the future research.

**FIGURE 2:**
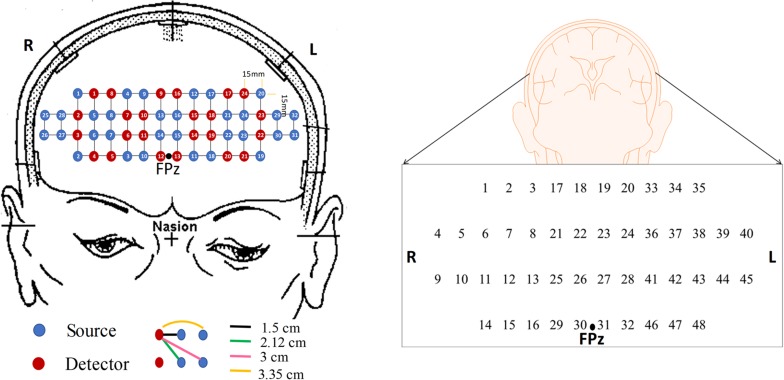
The configuration of the headset employed during the experiment, which consists of 24 emitters and 32 detectors **(left)** and the channels used in this paper **(right)**.

In this study, we investigate 15 digital biomarkers and 2 image biomarkers generated from the fNIRS hemodynamic responses for 15 MCI patients and 9 HC. The digital biomarkers take the form of mean, slope, peak, skewness, and kurtosis for a certain interval of time, and the two image biomarkers include *t*-map and correlation map. Finally, a conclusive result suggesting how to combine a biomarker and a classification method will be demonstrated, which turns out to be the combination of *t*-map and CNN classification. In the study, the used headset in Figure 2 covers the entire PFC (i.e., left PFC, middle PFC, and right PFC) making 204 channels. However, only 48 channels with sufficient emitter-detector distances (3 cm) are utilized. The performed three mental tasks include the *N-*back task, Stroop task, and VFT.

## Materials and Methods

### Participants

Twenty-four volunteers, who were right-handed and were able to communicate in Korean, were chosen for this study, comprising of fifteen patients with MCI (1 male and 14 females) and nine HC (2 males and 7 females) of similar age and educational background. MCI patients were recruited from the Pusan National University Hospital, Busan, Republic of Korea. The HC were selected from the local community on a voluntary basis. In addition, the mental state of each subject was examined using a Korean-Mini-Mental State Examination (K-MMSE), which is a 30-points questionnaire providing a quantitative measure of cognitive impairment (Han et al., 2008). The demographic information for all the volunteers including age (mean ± SD), gender, education background (mean ± SD), K-MMSE scores (mean ± SD), and statistical information are summarized in Table 2. This experiment was conducted in accordance with the latest Declaration of Helsinki upon the approval of the Pusan National University Institutional Review Board (General Assembly of the World Medical Association, 2014). All volunteers were given a detailed description of the experimental procedure prior to the beginning of the experiment, and they provided written consent agreeing to the experiments.

**TABLE 2:** The demographic information of all participants.

**Characteristics**	**MCI (*n* = 15)**	**HC (*n* = 9)**	***p*-value**
Gender (Male/Female)	1/14	2/7	0.44
Education [years]	11.2 (±4.81)	10.56 (±2.88)	0.36
Age [years]	69.27 (±7.09)	68.33 (±4.69)	0.36
K-MMSE Score	25.13 (±2.33)	27.22 (±1.98)	0.49

*K-MMSE, Korea Mini-Mental State Examination.*

### Channel Configuration

In this study, a near-infrared multi-channel continuous wave system (NIRSIT, OBELAB Inc., Rep. of Korea) with 8.138 Hz sampling rate was employed to measure the brain signals via 24 emitters and 32 detectors. The device has an active detection sensor with a total capacity of 204 channels out of which 48 channels were used in this study, which covered the entire PFC area. Channel 1 to channel 16 were placed in the right PFC, channel 17 to channel 32 in the middle PFC, and channel 33 to channel 48 in the left PFC. Figure 2 shows the locations of emitters and detectors with a reference point FPz (left) and the 48 channels in this study (right). The wavelengths used for detecting two chromophores (HbO, HbR) were 780 and 850 nm, respectively. As reported in Strangman et al. (2013), fNIRS is more sensitive to the gray matter and even a large source detector separation (up to ∼4.5 cm) can be used. Considering the spatial resolution and the differential path length factors into account, the pairs having the source detector distance of 3 cm were used.

### Experimental Paradigm

**FIGURE 3:**
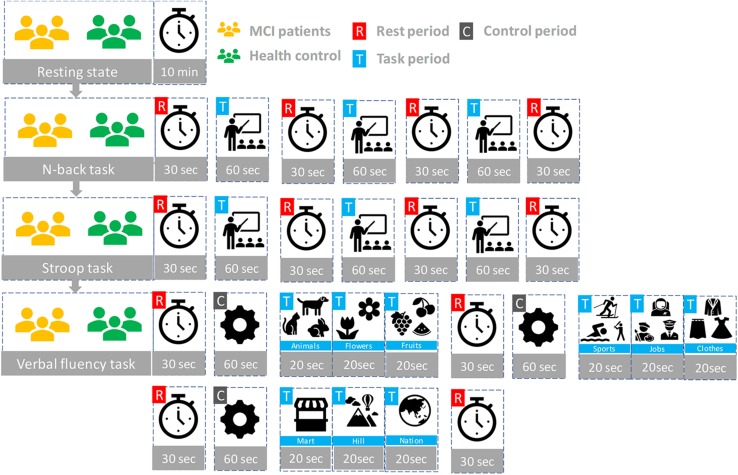
The experiment paradigms: Three tasks (*N*-back, Stroop, and verbal fluency) having three trials.

Participants seated on a comfortable chair and were instructed to avoid movement as much as possible. First, all subjects took a 10 min resting state. Subjects in each group participated in three sessions, which consisted of the *N-*back task, Stroop task, and semantic VFT. Each task took 60 s and was performed three times with a 30 s rest between tasks. Figure 3 illustrates the experimental paradigm for all three tasks. The *N-*back task evaluates the working memory (Kane et al., 2007) and, in our study, a two-back task was performed and one-digit numbers between 1–9 were displayed on the monitor. The subjects were asked to press the keyboard when the current number on the display matched the second-last number displayed before. The Stroop test is a measurement of widely used executive function and is known as a measurement of mental control and response flexibility. The Stroop task requires new reactions while suppressing the dominant response, such as letter reading conditions and color reading conditions, etc. In this study, the Korean-Color Word Stroop test (K-CWST) was used. The subjects were requested to read the color of letters when letters were written in red, blue, yellow, and black colors within a limited time (Byeon et al., 2017). The semantic VFT is a task to generate as many words (related to the given semantic category) as possible within a limited time (Whiteside et al., 2016). The task measures how much information can be retrieved from the categorization and memory repository of text for 1 min.

### Data Pre-processing

The fNIRS data were pre-processed and analyzed for each subject using MATLAB^TM^. The optical intensity signals were first transformed into the time series of HbO and HbR concentration changes using the modified Beer-Lambert law (MBLL) (Sassaroli and Fantini, 2004). The data were digitally bandpass-filtered to remove the physiological noises (respiration, cardiac activity, and low-frequency drift signals): For this, two fourth-order Butterworth filters (low and high-pass) with cutoff frequencies of 0.1 and 0.001 Hz, respectively, were used to filter off the noises from the converted hemodynamic signals (Khan and Hong, 2015, 2017). In this study, we analyzed both ΔHbO and ΔHbR signals for the evaluation of biomarkers, even though HbO signals are robust and more sensitive.

The previous comparison study between MCI and HC investigated by Li R. et al. (2018) indicated that utilizing the region of interest (ROI) strategy could provide the satisfying results with the averaged means and slope changes of ΔHbO. In this study, we implemented two strategies to identify the ROI; (i) *t*-value analysis and (ii) visual inspection (Privitera and Stark, 2000). In the first case, the active channels (i.e., *t* > 1.6469 and *p*-value < 0.05) were selected by using the MATLAB^TM^ function (*robustfit*), which becomes the ROI. In the second case, all the HbO signals were inspected visually, and those signals having the desired pattern were selected manually (i.e., visual inspection).

### Feature Extraction

Diverse biomarkers were evaluated, as a possible candidate, for an early identification of MCI. The considered digital biomarkers include the MHbO, SHbO, MHbR, SHbR, time to peak in the hemodynamic response, skewness, and kurtosis. In addition. we considered two image biomarkers: The *t*-map of all the channels and the correlation map of all the channels.

#### Digital Biomarkers

The HbO mean value change between the rest and task periods is represented as follows.

(1) $$M\,H\,b\,O=\frac{A\,v\,g\,\left(\Delta \,H\,b\,O_{t=t_{1}:t_{2}}\right)-A\,v\,g\,\left(\Delta \,H\,b\,O_{t=-10:0}\right)}{A\,v\,g\,\left(\Delta \,H\,b\,O_{t=-10:0}\right)}$$

where *t*_1_ and *t*_2_ are the starting and ending time in the selected time window, *t* = −10 indicates 10 s before the onset time, and *t* = 0 is the onset time of the task execution. The mean change of HbR concentration is computed as follows.

(2) $$M\,H\,b\,R=\frac{A\,v\,g\,\left(\Delta \,H\,b\,R_{t=t_{1}:t_{2}}\right)-A\,v\,g\,\left(\Delta \,H\,b\,R_{t=-10:0}\right)}{A\,v\,g\,\left(\Delta \,H\,b\,R_{t=-10:0}\right)}$$

We employed the *polyfit* function in MATLAB^TM^ to calculate the slope of HbO (i.e., SHbO) and the slope of HbR change (i.e., SHbR). The location of the peak, skewness, and kurtosis were conducted by using MATLAB^TM^ functions of *findpeaks*, *skewness*, and *kurtosis*, respectively.

#### Activation Map (*t*-Map)

To quantify cortical hemodynamic activities during the mental tasks, the general linear model (GLM, a model-based statistical analysis tool) was utilized (Pinti et al., 2017; Salis-Perales and Barajas-Ramirz, 2017). In GLM, the desired hemodynamic response function (dHRF) is used to serve as a reference to estimate the changes in HbO signals (Yennu et al., 2016). The formula is as follows:

(3) z⁢(t)=β⁢f⁢(t)+ε

(4) f⁢(t)=h⁢(t)⊗s⁢(t)

where *z*(*t*) represents the temporal profile of the measured ΔHbO or ΔHbR, β is the estimated amplitude of ΔHbO/ΔHbR, and ε represents the residual owing to the difference between the measured signals and the predicted model. *f*(*t*) is the stimulation-specific predicated response, which is expected to match the temporal profiles of the measured hemodynamic signal (i.e., dHRF); *h*(*t*) represent the canonical hemodynamic response function, and *s*(*t*) is the stimulation-specific boxcar function for a given task. Thus, after fitting equation (3), a statistical *t*-value representing a statistical significance of the brain activation with respect to the baseline at each respective channel was obtained. Moreover, the *t*-values were derived from *robustfit* for individual channels and were used to generate the *t*-map for a topographic image (Liu and Hong, 2017).

#### Channel-by-Channel Correlation Map

Comparing to fMRI, fNIRS has a significant advantage in temporal resolution. This advantage could provide convenience for investigating the functional connectivity of the prefrontal lobe by exploiting the temporal correlations channel by channel (Tak and Ye, 2014). The correlation map was calculated by using the MATLAB^TM^ correlation function (*corr*).

### Classification

In this study, the digital biomarkers were classified using the linear discriminant analysis (LDA) (Naseer et al., 2016) available as *classify* function in MATLAB^TM^. The tenfold cross validation method was used to estimate the classification performance of the predictive LDA model. The sample size in analyzing each digital biomarker becomes the number of subjects ***×*** the number of trials ***×*** the number channels in the ROI. The convolutional neural network (CNN) was utilized to conduct the classification of image biomarkers. CNN is highly capable of learning appropriate features automatically from the input data by optimizing the weight parameters in individual layer by using forward and backward propagation to minimize classification errors (Ding et al., 2017; Hamadache and Lee, 2017; Kim et al., 2017; Moon et al., 2018; Trakoolwilaiwan et al., 2019). The networks in this paper consist of four layers, including two convolutional layers and two fully connected layers. In the convolutional layers, a convolutional filter whose width is equal to the dimension of the input, and the kernel size of *h* is convolved with the input data, where the output of the *i*’th filter is represented as follows.

(5) $$output_{i}=w\cdot x\left[i:i+h-1\right]$$

(6) $$f\,\left(o\,u\,t\,p\,u\,t_{i}\right)=R\,e\,L\,U\,\left(o\,u\,t\,p\,u\,t_{i}\right)$$

(7) R⁢e⁢L⁢U⁢(x)=M⁢a⁢x⁢(o,x)

where *w* is the weights of the matrix and *x*[*i*:*j*] is the submatrix of the input from row *i* to *j*. Then the output of the first convolutional layer *f* (*output*) is converted by an activation function Re*LU*(*x*) to build the feature map. To enhance the performance, additional subsampling operation, max-pooling, and dropout (avoiding overfitting) are employed in this subsampling layer. To obtain an appropriate predictive model, the hyper-parameters such as the learning rate, batch size, and the number of epochs should be considered. In our study, the size of input data was 48 *×* 48. To maintain the original feature completely, we set up the batch size by 4. The grid search (Ou et al., 2019) and Adam optimization algorithm (β_1_ = 0.9, β_2_ = 0.1, and ε = 10^–8^; Tang et al., 2019) were utilized to choose the learning rate and the parameters in gradient descent optimization.

## Results

### Comparison of Hemodynamic Responses

**FIGURE 4:**
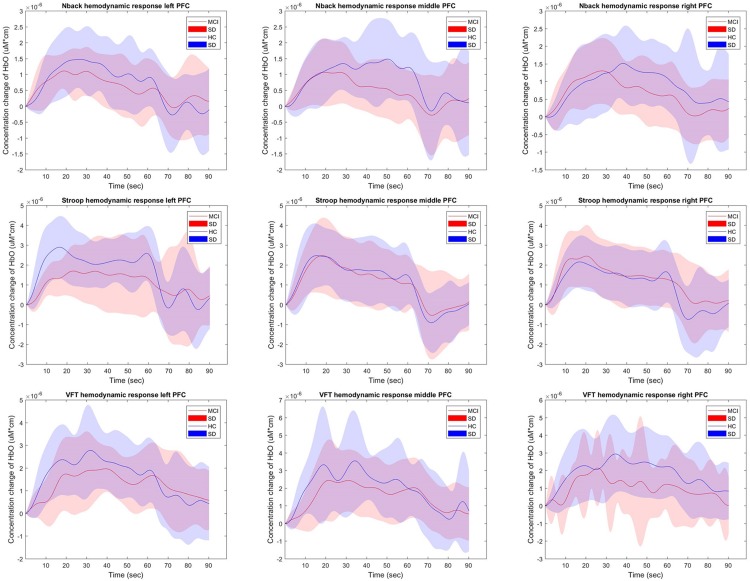
The average HbOs in three brain regions (**left, middle,** and **right** PFCs) of MCI patients and HC after performing three mental tasks.

Figure 4 shows the hemodynamic responses of ΔHbO from three brain regions (i.e., right, middle, and left PFC) of MCI patients and HC for three mental tasks (i.e., *N-*back task, Stroop task, and VFT). The purpose behind this strategy is to observe any visual differences between the MCI patients and HC. The figures plot the average HbOs of individual groups. MCI Patients are denoted by red color, whereas the corresponding SDs are shown with red shadows. HC are marked with blue color with its respective shadow in blue showing the SD. In the left brain region, the averaged concentration change of HbO for HC group is higher than that of MCI group in all three mental tasks. In addition, HC shows an earlier increase than MCI patients. But the middle and right PFCs do not show such a significant difference between two groups. The plots reveal that the brain regions have unique patterns of ΔHbO fluctuations. However, the averaged hemodynamic responses cannot tell the existence of improvement in cognition for the MCI patients, since their SDs were too large. Thus, the examination of the hemodynamic responses of ΔHbO is not sufficient to distinguish an individual from MCI or HC group. This leads us to the second technique, in which we will evaluate the digital biomarkers at using appropriate time intervals for statistical analysis.

### Statistical Analysis of Digital Biomarkers

To evaluate the digital biomarkers such as MHbO/MHbR, SHbO/SHbR, peak location, skewness, and kurtosis for the hemodynamic responses, we divided the PFC into three areas (i.e., left, middle and right PFC) and applied different time intervals for three mental tasks (i.e., *N-*back, Stroop and VFT), respectively. The statistical values of all the biomarkers obtained from the ROI channels (*t* > 1.6469) for three mental tasks are shown in Tables 3–5. Tables 6–8 present the statistical information of the biomarkers obtained from those channels selected by visual inspection. In this study, the task duration was set to 60 s. This is to see where the MCI patients can focus on the verbal fluency task for a somewhat long time period of time. Also, for comparison purposes, the task durations for *N*-back and Stroop tasks were to 60 s as well. The reason, why we considered the time period between 5 and 65 s, was due to the time delay (3–5 s) of the hemodynamic response (Naseer and Hong, 2015). The time interval of 5–25 s was selected since the initial peak time for hemodynamic response is nearly located in the first 20 s period. The slope features (i.e., SHbO/SHbR) were considered from three intervals of the hemodynamic response: First, the initial increasing interval of ΔHbO (i.e., from 5 to 15 s), the plateau period of ΔHbO during the task (i.e., from 20 to 60 s), and the final decreasing interval of ΔHbO (i.e., from 60 to 70 s). We expect that the MCI patients would have a light decline of ΔHbO during the second interval while performing the mental tasks if they cannot focus on the tasks, as seen in Figure 4. The time to peak (i.e., from 0 to the peak time) is to see when the peak value of the hemodynamic response occurs owing to the provided stimulation. Lastly, two biomarkers, skewness (from 5 to 65 s) and kurtosis (from 5 to 65 s) are to examine whether the overall profile of the hemodynamic responses of a MCI patient is different from that of HC. The entire biomarkers are summarized as follows.

**TABLE 3:** Statistical data of *N*-back task (based upon ROI channels).

**Biomarkers**		**Left PFC**			**Middle PFC**			**Right PFC**		
		**Avg.**	***SD***	***p*-value**	**Avg.**	***SD***	***p*-value**	**Avg.**	***SD***	***p*-value**
MHbO (5–65 s)	MCI	7.35e−07	4.80e−06	0.1511	1.29e−07	2.61e−06	0.3811	7.88e−07	2.92e−06	**0.0211**
	HC	4.37e−07	2.05e−06		8.25e−08	1.11e−06		4.59e−07	1.08e−06	
MHbR (5–65 s)	MCI	−2.56e−08	8.90e−07	0.6407	−6.60e−08	3.55e−07	0.9996	−1.69e−08	9.69e−07	0.0868
	HC	−1.18e−08	3.96e−07		−1.08e−08	2.00e−07		−7.02e−08	3.12e−07	
MHbO (5–25 s)	MCI	7.24e−07	3.67e−06	0.4381	3.01e−07	2.45e−06	0.4697	7.65e−07	2.91e−06	0.0491
	HC	6.87e−07	1.89e−06		2.90e−07	1.01e−06		5.06e−07	8.85e−07	
MHbR (5–25 s)	MCI	2.14e−08	1.33e−06	0.1062	−4.67e−08	5.53e−07	0.9690	8.33e−08	1.56e−06	**0.0148**
	HC	−5.17e−08	6.46e−07		5.10e−09	3.85e−07		−5.56e−08	5.39e−07	
MHbO (0–Peak seconds)	MCI	4.36e−07	5.61e−06	0.7752	2.32e−07	2.16e−06	0.6413	5.54e−07	2.41e−06	0.1252
	HC	5.61e−07	1.76e−06		2.75e−07	7.42e−07		4.08e−07	6.28e−07	
SHbO (5–15 s)	MCI	8.03e−09	6.11e−08	0.1840	2.33e−09	2.18e−08	0.6180	8.16e−09	4.52e−08	0.0283
	HC	4.55e−09	3.11e−08		2.77e−09	1.30e−08		3.50e−09	1.42e−08	
SHbR (5–15 s)	MCI	−1.09e−09	3.88e−08	0.3311	−2.43e−10	1.30e−08	0.3528	1.33e−09	4.40e−08	0.1437
	HC	−1.91e−09	2.49e−08		−4.61e−10	6.51e−09		−5.00e−10	1.06e−08	
SHbO (20–60 s)	MCI	−1.63e−09	1.46e−08	0.3163	−1.44e−09	3.51e−09	0.4562	−1.35e−09	1.28e−08	0.8269
	HC	−2.07e−09	7.31e−09		−1.20e−09	3.74e−09		−6.80e−10	4.88e−09	
SHbR (20–60 s)	MCI	4.29e−10	1.11e−08	0.4641	1.32e−10	2.45e−09	0.2921	−3.01e−10	1.24e−08	0.7714
	HC	3.88e−10	3.84e−09		1.22e−11	4.13e−09		7.21e−11	3.94e−09	
SHbO (60–70 s)	MCI	−1.68e−08	1.07e−07	0.4975	−1.13e−08	2.57e−08	0.0842	−1.84e−08	6.49e−08	0.8426
	HC	−1.69e−08	3.62e−08		−1.39e−08	1.96e−08		−1.49e−08	2.29e−08	
SHbR (60–70 s)	MCI	1.50e−09	8.36e−08	0.3460	2.44e−09	2.96e−08	0.0605	−6.23e−10	6.01e−08	0.7577
	HC	5.73e−11	3.96e−08		1.81e−10	1.97e−08		1.13e−09	2.31e−08	
SHbO (0–Peak seconds)	MCI	1.81e−08	6.91e−08	0.2161	8.80e−09	2.65e−08	0.0842	1.97e−08	5.82e−08	**0.0011**
	HC	1.46e−08	3.75e−08		9.16e−09	1.58e−08		1.03e−08	1.51e−08	
Peak time (seconds)	MCI	1.50e + 01	5.62e + 00	0.8265	1.47e + 01	4.93e + 00	**0.0245**	1.56e + 01	5.36e + 00	0.1738
	HC	1.51e + 01	5.36e + 00		1.38e + 01	5.53e + 00		1.50e + 01	5.36e + 00	
Skewness	MCI	−8.02e−02	6.42e−01	0.3731	−1.05e−01	6.65e−01	0.5418	−1.66e−01	6.45e−01	**0.0015**
	HC	−1.01e−01	7.61e−01		−9.73e−02	8.99e−01		−3.52e−01	8.40e−01	
Kurtosis	MCI	2.26e + 00	9.64e−01	0.4155	2.18e + 00	8.96e−01	0.5462	2.19e + 00	7.74e−01	0.9987
	HC	2.33e + 00	9.81e−01		2.23e + 00	8.77e−01		2.40e + 00	8.70e−01	

**TABLE 4:** Statistical data of Stroop task (based upon ROI channels).

**Biomarkers**		**Left PFC**			**Middle PFC**			**Right PFC**		
		**Avg.**	***SD***	***p*-value**	**Avg.**	***SD***	***p*-value**	**Avg.**	***SD***	***p*-value**
MHbO (5–65 s)	MCI	8.43e−07	3.65e−06	0.5778	4.15e−07	2.44e−06	0.0644	9.03e−07	3.51e−06	0.3884
	HC	9.26e−07	4.72e−06		8.50e−07	2.43e−06		8.30e−07	2.78e−06	
MHbR (5–65 s)	MCI	3.96e−07	9.66e−07	0.9089	2.06e−07	4.42e−07	0.1575	3.98e−07	8.00e−07	0.1772
	HC	4.07e−07	9.07e−07		2.65e−07	4.45e−07		3.32e−07	6.49e−07	
MHbO (5–25 s)	MCI	1.05e−06	3.47e−06	0.6053	7.23e−07	2.41e−06	**0.0298**	1.21e−06	3.97e−06	0.1971
	HC	1.16e−06	4.89e−06		1.21e−06	2.15e−06		9.85e−07	2.39e−06	
MHbR (5–25 s)	MCI	2.49e−07	1.36e−06	0.7795	1.70e−07	5.45e−07	0.5652	2.90e−07	1.21e−06	0.1860
	HC	3.37e−07	1.05e−06		1.99e−07	4.88e−07		2.08e−07	6.92e−07	
MHbO (0–Peak seconds)	MCI	9.37e−07	2.99e−06	0.3400	5.44e−07	1.71e−06	**0.0057**	8.78e−07	3.13e−06	0.3101
	HC	8.00e−07	3.63e−06		9.78e−07	1.39e−06		7.80e−07	1.77e−06	
SHbO (5–15 s)	MCI	3.27e−09	5.52e−08	0.9798	6.83e−09	2.78e−08	0.0919	1.81e−08	9.39e−08	**0.0083**
	HC	1.16e−08	3.37e−08		4.03e−09	1.87e−08		6.03e−09	2.06e−08	
SHbR (5–15 s)	MCI	8.57e−09	4.05e−08	**0.0344**	1.98e−09	1.54e−08	0.1172	1.49e−08	9.84e−08	0.0192
	HC	3.10e−09	2.37e−08		−4.08e−10	1.70e−08		2.39e−09	1.51e−08	
SHbO (20–60 s)	MCI	−1.33e−09	9.82e−09	0.1610	−2.08e−09	6.75e−09	0.6976	−1.82e−09	1.71e−08	0.9298
	HC	2.04e−11	1.04e−08		−1.81e−09	4.44e−09		−4.28e−10	5.18e−09	
SHbR (20–60 s)	MCI	3.37e−10	8.87e−09	0.8996	−8.35e−11	2.48e−09	**0.0149**	5.42e−10	9.60e−09	0.2607
	HC	2.33e−10	7.89e−09		4.84e−10	2.46e−09		1.19e−10	4.00e−09	
SHbO (60–70 s)	MCI	−3.14e−08	1.01e−07	**0.0045**	−3.04e−08	7.63e−08	**0.0061**	−2.70e−08	9.68e−08	**0.0029**
	HC	−6.23e−08	1.32e−07		−5.83e−08	1.27e−07		−5.13e−08	1.10e−07	
SHbR (60–70 s)	MCI	−2.22e−08	7.17e−08	0.7094	−7.05e−09	1.71e−08	**0.0014**	−2.18e−08	6.91e−08	0.8339
	HC	−1.88e−08	5.78e−08		−1.40e−08	2.93e−08		−1.65e−08	4.36e−08	
SHbO (0–Peak seconds)	MCI	2.69e−08	7.96e−08	0.0859	1.37e−08	4.30e−08	0.9788	2.81e−08	7.13e−08	**0.0008**
	HC	1.87e−08	5.08e−08		2.08e−08	3.32e−08		1.48e−08	3.07e−08	
Peak time (seconds)	MCI	1.41e + 01	5.80e + 00	0.0775	1.51e + 01	5.85e + 00	0.9238	1.47e + 01	5.60e + 00	0.2485
	HC	1.50e + 01	5.21e + 00		1.52e + 01	6.43e + 00		1.52e + 01	5.76e + 00	
Skewness	MCI	−1.36e−01	8.97e−01	**0.0045**	−6.57e−02	8.64e−01	**0.0032**	−3.61e−02	8.88e−01	**0.0002**
	HC	−4.06e−01	1.14e + 00		−3.53e−01	1.17e + 00		−3.43e−01	1.13e + 00	
Kurtosis	MCI	2.48e + 00	1.06e + 00	0.9423	2.48e + 00	1.07e + 00	0.6990	2.52e + 00	1.17e + 00	0.3557
	HC	2.67e + 00	1.26e + 00		2.44e + 00	1.06e + 00		2.43e + 00	1.15e + 00	

**TABLE 5:** Statistical data of VFT (based upon ROI channels).

**Biomarkers**		**Left PFC**			**Middle PFC**			**Right PFC**		
		**Avg.**	***SD***	***p*-value**	**Avg.**	***SD***	***p*-value**	**Avg.**	***SD***	***p*-value**
MHbO (5–65 s)	MCI	9.54e−07	4.05e−06	0.7156	3.04e−06	3.04e−06	0.8332	1.34e−06	5.09e−06	0.6294
	HC	1.09e−06	2.06e−06		1.04e−06	2.39e−06		1.43e−06	2.09e−06	
MHbR (5–65 s)	MCI	3.52e−07	9.62e−07	**0.0032**	1.55e−07	4.80e−07	0.0907	3.10e−07	8.44e−07	**0.0000**
	HC	1.75e−07	3.74e−07		1.08e−07	2.45e−07		1.03e−07	2.39e−07	
MHbO (5–25 s)	MCI	9.27e−07	3.21e−06	0.8961	3.07e−06	3.07e−06	0.9405	1.29e−06	5.49e−06	0.6196
	HC	1.20e−06	2.37e−06		1.21e−06	2.49e−06		1.39e−06	1.85e−06	
MHbR (5–25 s)	MCI	2.65e−07	1.38e−06	0.5851	2.23e−07	8.54e−07	0.2009	3.34e−07	1.82e−06	0.1186
	HC	2.87e−07	7.17e−07		1.73e−07	3.87e−07		1.98e−07	5.84e−07	
MHbO (0–Peak seconds)	MCI	7.63e−07	2.59e−06	0.6852	2.63e−06	2.63e−06	0.7594	7.56e−07	4.85e−06	0.8759
	HC	8.44e−07	1.78e−06		8.40e−07	1.86e−06		1.06e−06	1.53e−06	
SHbO (5–15 s)	MCI	6.80e−09	1.04e−07	0.6619	5.04e−08	5.04e−08	0.9667	1.93e−08	1.25e−07	**0.0393**
	HC	9.12e−09	2.77e−08		1.12e−08	3.22e−08		7.43e−09	3.61e−08	
SHbR (5–15 s)	MCI	1.08e−08	5.56e−08	**0.0347**	3.82e−09	2.71e−08	0.2267	1.58e−09	1.09e−07	0.4574
	HC	3.37e−09	2.98e−08		2.37e−09	1.33e−08		8.90e−10	1.30e−08	
SHbO (20–60 s)	MCI	8.45e−10	1.26e−08	**0.0002**	1.12e−08	1.12e−08	0.4277	−1.08e−10	2.02e−08	0.1714
	HC	−1.92e−09	7.42e−09		−1.70e−09	4.83e−09		−1.15e−09	6.04e−09	
SHbR (20–60 s)	MCI	−1.42e−11	1.01e−08	0.0922	−5.89e−10	4.50e−09	0.1039	7.31e−10	1.47e−08	**0.044**
	HC	−9.76e−10	5.00e−09		−9.98e−10	2.24e−09		−8.23e−10	3.83e−09	
SHbO (60–70 s)	MCI	−1.65e−08	7.67e−08	0.8321	1.12e−08	4.82e−08	0.429	−2.18e−08	9.44e−08	0.6000
	HC	−1.22e−08	3.45e−08		−6.72e−09	3.22e−08		−2.04e−08	4.50e−08	
SHbR (60–70 s)	MCI	−1.75e−08	6.75e−08	0.9888	−2.68e−09	1.46e−08	0.1605	−1.01e−08	8.96e−08	0.8797
	HC	−7.53e−09	2.23e−08		−4.73e−09	1.42e−08		−3.83e−09	1.46e−08	
SHbO (0–Peak seconds)	MCI	2.41e−08	7.05e−08	0.1679	4.82e−08	3.55e−08	0.9989	4.05e−08	1.25e−07	**0.0098**
	HC	2.02e−08	3.02e−08		2.34e−08	2.68e−08		2.44e−08	3.99e−08	
Peak time (seconds)	MCI	1.54e + 01	5.55e + 00	0.3332	5.68e + 00	5.68e + 00	**0.0051**	1.56e + 01	5.20e + 00	0.0624
	HC	1.50e + 01	5.44e + 00		1.40e + 01	5.73e + 00		1.47e + 01	5.66e + 00	
Skewness	MCI	−4.01e−02	6.38e−01	0.6858	6.97e−01	6.97e−01	0.9286	−7.38e−02	7.17e−01	0.2114
	HC	3.08e−02	6.86e−01		−2.59e−02	7.78e−01		−1.47e−01	7.29e−01	
Kurtosis	MCI	2.29e + 00	9.39e−01	0.3276	2.28e + 00	1.08e + 00	0.4949	2.29e + 00	1.02e + 00	0.2745
	HC	2.36e + 00	9.26e−01		2.28e + 00	7.88e−01		2.25e + 00	8.25e−01	

**TABLE 6:** Statistical data of *N*-back task (manually selected channels).

**Biomarkers**		**Left PFC**			**Middle PFC**			**Right PFC**		
		**Avg.**	***SD***	***p*-value**	**Avg.**	***SD***	***p*-value**	**Avg.**	***SD***	***p*-value**
MHbO (5–65 s)	MCI	7.99e−07	6.54e−07	0.0827	6.44e−07	6.76e−07	**0.0108**	7.78e−07	5.65e−07	0.5999
	HC	1.05e−06	6.93e−07		9.70e−07	7.08e−07		8.35e−07	6.62e−07	
MHbO (5–25 s)	MCI	9.23e−07	6.23e−07	0.7490	8.25e−07	7.61e−07	0.2285	8.16e−07	5.81e−07	**0.0047**
	HC	1.03e−06	9.00e−07		7.03e−07	9.99e−07		4.85e−07	8.46e−07	
MHbO (0–Peak seconds)	MCI	6.62e−07	4.92e−07	0.9820	8.25e−07	5.20e−07	0.1477	4.94e−07	4.81e−07	**0.0056**
	HC	6.65e−07	7.56e−07		3.64e−07	8.60e−07		2.00e−07	6.65e−07	
SHbO (5–15 s)	MCI	5.94e−09	8.40e−09	**0.0025**	8.59e−09	1.20e−08	0.5788	1.06e−08	1.33e−08	0.7049
	HC	1.24e−08	1.04e−08		9.79e−09	1.15e−08		9.71e−09	1.29e−08	
SHbO (20–60 s)	MCI	−2.45e−09	2.58e−09	0.4853	−2.52e−09	3.58e−09	**0.0002**	−2.49e−09	3.82e−09	**0.0002**
	HC	−2.48e−09	4.16e−09		3.22e−10	4.40e−09		3.00e−10	4.41e−09	
SHbO (60–70 s)	MCI	−6.43e−09	1.41e−08	**0.0128**	−8.51e−09	1.75e−08	**0.0040**	−7.55e−09	1.46e−08	0.7138
	HC	−1.58e−08	1.90e−08		−1.83e−08	1.91e−08		−5.36e−09	2.86e−08	
SHbO (0–Peak seconds)	MCI	1.28e−08	1.09e−08	0.7124	1.29e−08	9.43e−09	0.4330	1.29e−08	8.34e−09	0.1379
	HC	1.20e−08	8.63e−09		1.15e−08	9.47e−09		1.04e−08	1.01e−08	
Peak time (seconds)	MCI	1.65e + 01	5.07e + 00	0.1711	1.62e + 01	4.90e + 00	0.5788	1.61e + 01	5.16e + 00	0.2327
	HC	1.79e + 01	4.83e + 00		1.66e + 01	5.36e + 00		1.72e + 01	5.03e + 00	
Skewness	MCI	−8.92e−02	6.39e−01	0.6745	−9.64e−02	6.60e−01	0.1374	−7.30e−02	6.90e−01	**0.0067**
	HC	−3.88e−02	4.93e−01		−2.95e−01	7.98e−01		−4.37e−01	7.91e−01	
Kurtosis	MCI	2.28e + 00	9.35e−01	0.3903	2.26e + 00	1.05e + 00	**0.0121**	2.32e + 00	8.19e−01	0.7654
	HC	2.46e + 00	9.53e−01		2.73e + 00	9.78e−01		2.44e + 00	1.06e + 00	

**TABLE 7:** Statistical data of Stroop task (manually selected channels).

**Biomarkers**		**Left PFC**			**Middle PFC**			**Right PFC**		
		**Avg.**	***SD***	***p*-value**	**Avg.**	***SD***	***p*-value**	**Avg.**	***SD***	***p*-value**
MHbO (5–65 s)	MCI	1.40e−06	1.33e−06	**0.0129**	1.60e−06	1.39e−06	0.6469	1.68e−06	1.23e−06	0.5993
	HC	2.27e−06	1.29e−06		1.75e−06	1.44e−06		1.52e−06	1.24e−06	
MHbO (5–25 s)	MCI	1.27e−06	8.05e−07	0.9999	2.00e−06	1.50e−06	0.7069	2.10e−06	1.29e−06	0.2326
	HC	2.47e−06	1.42e−06		2.13e−06	1.48e−06		1.76e−06	1.10e−06	
MHbO (0–Peak seconds)	MCI	9.21e−07	6.42e−07	**0.0009**	1.42e−06	1.04e−06	0.7069	1.46e−06	9.74e−07	0.2140
	HC	1.67e−06	1.01e−06		1.51e−06	1.08e−06		1.20e−06	8.01e−07	
SHbO (5–15 s)	MCI	1.27e−08	1.38e−08	0.0555	2.13e−08	1.85e−08	0.9113	1.97e−08	1.75e−08	0.3121
	HC	1.99e−08	1.47e−08		2.09e−08	1.53e−08		1.81e−08	1.22e−08	
SHbO (20–60 s)	MCI	−9.36e−10	4.49e−09	0.3268	−3.66e−09	3.27e−09	0.1450	−3.00e−09	3.49e−09	0.9623
	HC	7.47e−11	3.22e−09		−2.64e−09	2.50e−09		−1.81e−09	2.25e−09	
SHbO (60–70 s)	MCI	−8.95e−09	2.01e−08	**0.0000**	−2.19e−08	3.76e−08	**0.0471**	−1.17e−08	1.82e−08	**0.0001**
	HC	−3.98e−08	1.74e−08		−3.27e−08	2.05e−08		−3.43e−08	2.62e−08	
SHbO (0–Peak seconds)	MCI	1.53e−08	9.22e−09	0.9999	2.26e−08	1.57e−08	0.8587	2.67e−08	1.83e−08	0.2631
	HC	3.29e−08	2.12e−08		2.78e−08	2.34e−08		2.23e−08	1.43e−08	
Peak time (seconds)	MCI	1.82e + 01	6.33e + 00	**0.0104**	1.71e + 01	4.07e + 00	0.6153	1.68e + 01	5.41e + 00	0.9528
	HC	1.50e + 01	3.67e + 00		1.76e + 01	5.51e + 00		1.68e + 01	4.61e + 00	
Skewness	MCI	−4.56e−01	7.31e−01	0.3755	−1.09e−01	9.53e−01	0.1087	−2.62e−01	9.96e−01	0.7460
	HC	−2.90e−01	7.03e−01		−4.49e−01	8.28e−01		−3.34e−01	9.03e−01	
Kurtosis	MCI	3.00e + 00	1.32e + 00	0.6991	2.53e + 00	9.36e−01	0.6885	2.61e + 00	1.11e + 00	0.6029
	HC	3.14e + 00	1.50e + 00		2.63e + 00	1.23e + 00		2.47e + 00	1.32e + 00	

**TABLE 8:** Statistical data of VFT (manually selected channels).

**Biomarkers**		**Left PFC**			**Middle PFC**			**Right PFC**		
		**Avg.**	***SD***	***p*-value**	**Avg.**	***SD***	***p*-value**	**Avg.**	***SD***	***p*-value**
MHbO (5–65 s)	MCI	1.56e−06	9.50e−07	**0.0371**	1.60e−06	1.39e−06	0.6469	1.24e−06	7.60e−07	1.0000
	HC	2.12e−06	1.06e−06		1.75e−06	1.44e−06		2.21e−06	1.59e−06	
MHbO (5–25 s)	MCI	1.09e−06	1.26e−06	**0.0004**	2.00e−06	1.50e−06	0.7069	1.18e−06	9.32e−07	0.9934
	HC	2.29e−06	1.23e−06		2.13e−06	1.48e−06		1.82e−06	1.62e−06	
MHbO (0–Peak seconds)	MCI	7.33e−07	8.93e−07	**0.0001**	1.42e−06	1.04e−06	0.7069	6.98e−07	5.18e−07	0.9993
	HC	1.79e−06	9.83e−07		1.51e−06	1.08e−06		1.24e−06	1.11e−06	
SHbO (5–15 s)	MCI	8.50e−09	1.99e−08	0.2225	2.13e−08	1.85e−08	0.9113	1.97e−08	2.29e−08	0.8179
	HC	1.41e−08	1.42e−08		2.09e−08	1.53e−08		1.84e−08	2.74e−08	
SHbO (20–60 s)	MCI	−7.78e−10	5.29e−09	0.1965	−3.66e−09	3.27e−09	0.1450	−3.39e−09	7.52e−09	0.9903
	HC	−2.42e−09	4.26e−09		−2.64e−09	2.50e−09		−9.40e−11	5.40e−09	
SHbO (60–70 s)	MCI	−4.39e−09	2.14e−08	0.0597	−2.19e−08	3.76e−08	**0.0471**	−6.18e−09	1.40e−08	**0.0126**
	HC	−1.37e−08	1.49e−08		−3.27e−08	2.05e−08		−1.45e−08	1.73e−08	
SHbO (0–Peak seconds)	MCI	1.26e−08	1.46e−08	**0.0110**	2.26e−08	1.57e−08	0.8587	1.46e−08	1.91e−08	**0.0145**
	HC	2.34e−08	1.74e−08		2.78e−08	2.34e−08		2.55e−08	2.30e−08	
Peak time (seconds)	MCI	1.84E + 01	4.47E + 00	0.9834	1.71E + 01	4.07E + 00	0.6153	1.71E + 01	4.07E + 00	0.4011
	HC	1.84E + 01	3.76E + 00		1.76E + 01	5.51E + 00		1.76E + 01	5.51E + 00	
Skewness	MCI	−6.96e−02	5.60e−01	0.7028	−1.09e−01	9.53e−01	0.1087	1.55e−02	4.91e−01	**0.0409**
	HC	−1.22e−01	4.90e−01		−4.49e−01	8.28e−01		−2.31e−01	6.38e−01	
Kurtosis	MCI	2.12E + 00	6.62e−01	0.9957	2.53E + 00	9.36e−01	0.6885	1.90E + 00	1.04E + 00	0.9623
	HC	2.75E + 00	1.05E + 00		2.63E + 00	1.23E + 00		2.25E + 00	7.70e−01

Biomarker 1: MHbO in the interval of 5∼65 s
Biomarker 2: MHbR in the interval of 5∼65 s
Biomarker 3: MHbO in the interval of 5∼25 s
Biomarker 4: MHbR in the interval of 5∼25 s
Biomarker 5: MHbO in the interval of 0 – Peak
Biomarker 6: SHbO from 5 to 15 s
Biomarker 7: SHbR from 5 to 15 s
Biomarker 8: SHbO from 20 to 60 s
Biomarker 9: SHbR from 20 to 60 s
Biomarker 10: SHbO from 60 to 70 s
Biomarker 11: SHbR from 60 to 70 s
Biomarker 12: SHbO from 0 to the peak
Biomarker 13: Peak time of ΔHbO
Biomarker 14: Skewness of ΔHbO for the duration of 5 – 65 s
Biomarker 15: Kurtosis of ΔHbO for the duration of 5 – 65 s

This study employed two-sample independent *t*-test to conduct the statistical analysis with the significance level of 0.05. The *p*-value lower than 0.05 indicates the existence of significance difference between two groups. As demonstrated in Tables 3–8, the biomarkers with *p*-value < 0.05 are considered as ones with significant differences between the MCI and HC groups, which are marked bold. The appearance of significant biomarkers was random. It is remarked that the obtained biomarkers were not repeated for all three tasks or brain regions. Only Biomarker 14 (skewness) revealed a significant difference for all three brain regions when performing the Stroop task in the case of ROI channels, see Table 4. Although a few biomarkers showed some difference (similarly to Section “Comparison of Hemodynamic Responses”), the group statistical analysis was difficult to permit a meaningful diagnostic result for the individuals. This leads to our third strategy, Section “Classification of Digital Biomarkers”: Evaluating the individual classification accuracy using digital biomarkers for three mental tasks and different brain regions.

### Classification of Digital Biomarkers

**FIGURE 5:**
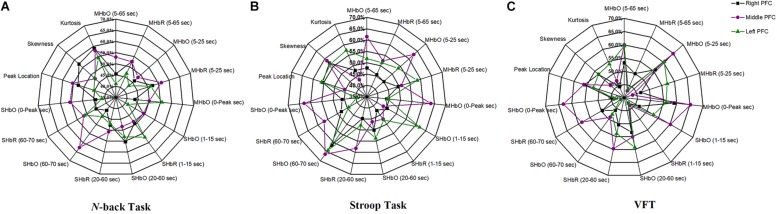
Panels **(A–C)** are the LDA classification results of 15 digital biomarkers based upon ROI channels with N-back, Stroop, and VFT task, respectively.

**FIGURE 6:**
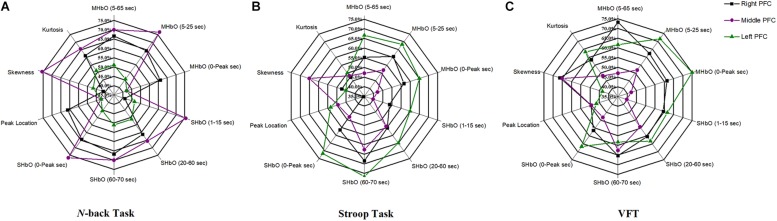
Panels **(A–C)** are the LDA classification results of 10 digital biomarkers based upon manually selected ROI channels with N-back, Stroop, and VFT task, respectively.

The selection of the time intervals for Biomarkers 1–15 has already been discussed in Section “Statistical Analysis of Digital Biomarkers.” Figure 5 depicts the entire classification accuracies between MCI and HC based upon ROI channels for (i) three mental tasks, (ii) three brain regions, and (iii) fifteen biomarkers. LDA was used as a classifier. On the other hand, for the channels manually selected, Figure 6 shows the comparative data for Figure 5. In agreement with the previous results (Yap et al., 2017), ΔHbO shows better classification results compared to ΔHbR (see Biomarkers 1 and 2 of *N*-back and Stoop tasks in Figures 5A,B). Therefore, in the case of manually selected channels, the analysis of ΔHbR is omitted and only ΔHbO is focused. Surprisingly, Biomarker 1 in the middle PFC when channels were selected manually showed the higher classification result than the case of ROI channels. It is remarked that the biomarkers showing significant difference in Tables 3–8 are not necessarily going to have the same satisfactory classification results with LDA: For instance, Biomarkers 6 and 10 showed a good classification results in Table 6, but it is not so in Figure 6A. Even though the best accuracy of 76.67% (i.e., Biomarker 11 of *N*-back task and Biomarker 10 of Stroop task) was achieved by using LDA, it is still considered low to be implemented for clinical applications. As previously mentioned, these 15 biomarkers were chosen based on the existing studies and our own experiences. But, the low classification result using LDA necessitates a further pursuit toward a reliable biomarker for MCI patients based on the hemodynamic response. We therefore consider using the whole or selected hemodynamic responses in combination with a machine learning method, CNN.

### CNN Classification of Hemodynamic Responses

**FIGURE 7:**
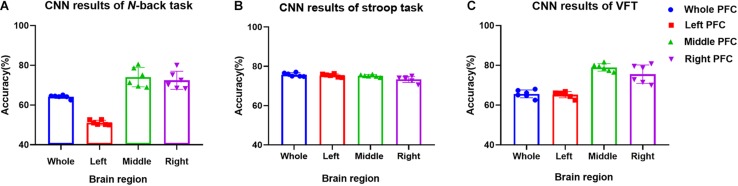
Classification results by CNN (repeated six times) using the HbO data in the ROI.

In this section, we investigate the CNN method for automatic learning of the useful features from the hemodynamic responses between MCI and HC. We regard that most of the valuable features appearing in the digital biomarkers are already contained in the non-linear feature form in the CNN model. As demonstrated in Figure 7, the CNN classification results trained by the concentration changes of the ΔHbO of the *N-*back task show approximately similar accuracies in the three brain regions (i.e., whole PFC: 64.21%, right PFC: 72.46%, and middle PFC 74.03%) except for the left PFC, which has the lowest accuracy than other regions. The classification accuracies with the Stroop task ranged from a minimum of 73.36% (right PFC) to a maximum of 75.77% (left PFC). In the VFT case, the middle PFC obtained a good classification accuracy (78.94%) in comparison to the whole PFC, left PFC, and right PFC. The classification accuracies were improved in comparison to the LDA results obtained by the digital biomarkers. Even the best accuracy in the case of CNN results trained by hemodynamic response was nearly 80% (i.e., 78.94% in Figure 7C), the potential to increase the accuracy still exists. To push the boundary for a better classification accuracy, we employ the *t*-map and the correlation map as biomarkers for classifying the MCI patients from HC.

### CNN Classification Results of Imaging Biomarkers

**FIGURE 8:**
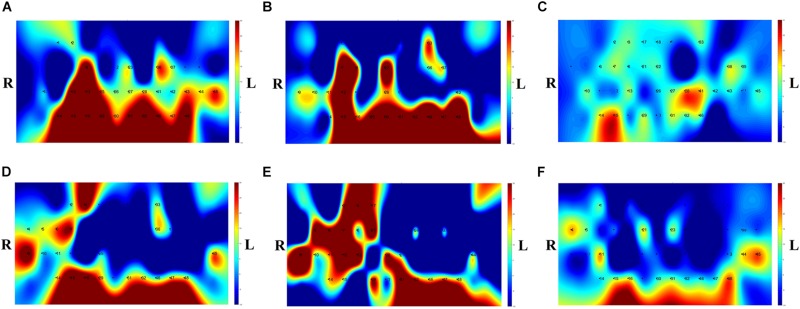
Comparison of average *t*-maps between MCI and HC for three mental tasks: **(A)** *N*-back MCI, **(B)** Stroop MCI, **(C)** VFT MCI, **(D)** *N*-back HC, **(E)** Stroop HC, and **(F)** VFT HC.

**FIGURE 9:**
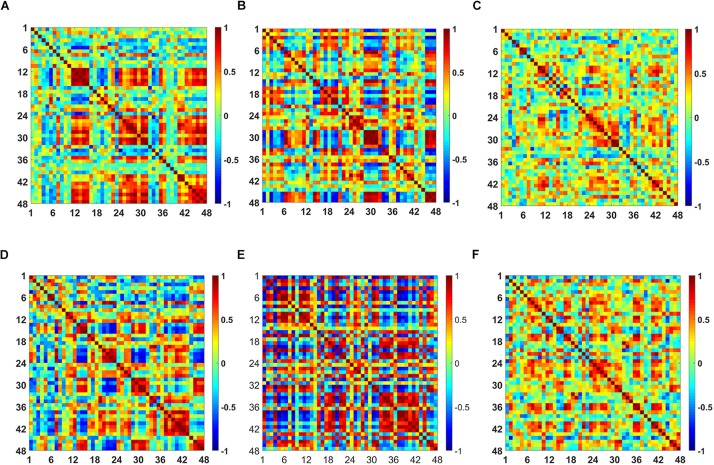
Comparison of the correlation maps between MCI and HC for three mental tasks: **(A)** *N*-back MCI, **(B)** Stroop MCI, **(C)** VFT MCI, **(D)** *N*-back HC, **(E)** Stroop HC, and **(F)** VFT HC (the color bar in the right shows the correlation coefficient from –1 to 1).

**FIGURE 10:**
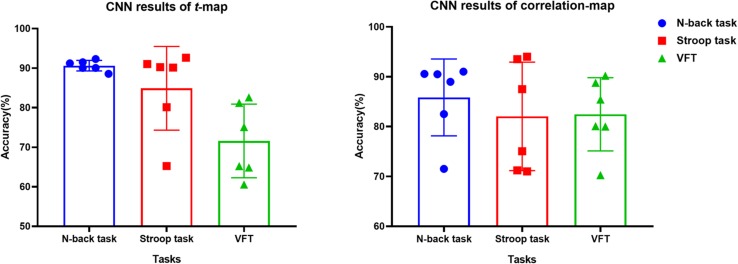
Classification results of *t*-maps and correlation maps by CNN (repeated six times) for three mental tasks.

The *t*-map and correlation map are widely used as an image biomarker in the field of fMRI. Figure 8 shows the group averaged *t*-maps of three mental tasks. The numbers shown in Figure 8 represent the channel numbers on the PFC. The top three figures in Figure 8 (i.e., A–C) present the *t*-maps generated by MCI group with the *N-*back task, Stroop task, and VFT, respectively, and the lower three maps represent those of HC (i.e., Figures 8D–F). The results reveal that the activated regions between MCI patients and HC are different. Figure 9, portrays the correlation maps of three mental tasks for MCI (Figures 9A–C) and HC (Figures 9D–F). Finally, the CNN results trained by *t*-map and correlation map are compared in Figure 10. All the CNN results (accuracy) trained by both image biomarkers were higher than 82.05%, except for the VFT task and *t*-map (71.59%). Particularly, the CNN result trained by *t*-map with the *N-*back task showed a highest accuracy of 90.62%.

## Discussion

In this paper, our goal is to propose the best biomarker for diagnosing the MCI patients for clinical usage. For this, 15 digital biomarkers (5 means and 7 slopes of ΔHbO/ΔHbR, peak time, skewness, kurtosis), three PFC regions, and two image biomarkers (*t*-map, correlation map) were investigated for detecting neural degeneration in the MCI patients. This study also aims at developing a novel method for diagnosing the MCI patients from the elderly in their everyday environment using fNIRS. To the best of the authors’ knowledge, this is the first work for evaluating the digital biomarkers in relation to MCI/AD with fNIRS. The obtained results can become a reference for utilizing appropriate biomarkers for neural information detection, and may provide a new tool to diagnose MCI patients in a harmless, non-invasive and portable manner.

(i) Statistical analysis and individual classification: In Figure 4 and Tables 3–8, the existence of differences of hemodynamic responses between two groups (MCI, HC) is shown. Most biomarkers in Tables 3–8 as well as the differences in HbOs in Figure 4 reveal the existence. This is consistent with the former studies (Katzorke et al., 2017; Vermeij et al., 2017; Yap et al., 2017; Li R. et al., 2018). However, the LDA classification accuracies based up the biomarkers shown in Figure 5 are too low for clinical applications. That means that the statistical analysis approach is not reliable for the detection of an MCI patient clinically. Beyond the current method, a new method of using the averaged hemodynamic responses of MCI patients and HC should be investigated, for instance, adaptive estimation algorithms (Iqbal et al., 2018; Nguyen et al., 2018; Yazdani et al., 2018; Yi et al., 2018) or advanced signal processing (Chen et al., 2018; Hong et al., 2018a).

(ii) Better results in local PFCs: In the literature, Goh and Park (2009) proposed the scaffolding theory for aging and cognition. Similar results (Cabeza et al., 2002; Katzorke et al., 2018) also verified that a neural compensatory mechanism exists and an additional neural passageway is recruited to support the declining brain function if it becomes inefficient. Similar with this compensation theory, the ΔHbO of HC in the left PFC (shown in Figure 3) appeared higher than that of MCI, but this was not obvious in the right and middle PFCs. This result is consistent with the work of Reuter-Lorenz et al. (2000), which claims that the contralateral right PFC of the patients with MCI can increase recruitment of both working memory and episodic encoding. Also, the higher classification result when using the middle PFC, as seen in Figure 7, indicates that the middle brain activity got decreased in the MCI patients. This may coincide with the fact that the gray matter in the middle PFC gets reduced during the process of aging (Minkova et al., 2017).

(iii) ROI strategy: Two strategies for selecting the signals for analysis were evaluated; *t*-value based selection and manual selection by visual examination. The *t*-value based ROI selection is widely employed in the bio-signal processing areas (Plichta et al., 2006), since it has the advantage of being convenient and consumes lesser time. However, in this study, we found that the automatic ROI selection with *t* > *t*_crit_ included many data with high noise oscillations. As revealed in Figures 5, 6 and Tables 3–8, the results obtained by using the manually selected active channels showed a better performance than the automatic ROI selection. It reveals that the channel selection is very sensitive to the final result because the poor performance could be caused by the wrong selection of ROI channels algorithmically. In light of the above-mentioned advantage, the automatic ROI selection would be convenient when analyzing a big data set.

(iv) Mental tasks: Three mental tasks (*N*-back, Stroop, VFT) were employed to classify the MCI patients from HC. Based on the hemodynamic response of ΔHbO, the statistical digital biomarkers analysis, and digital/image biomarkers classification, the *N-*back task showed a robust and stable performance in contrast to the Stroop task and VFT. Especially, the CNN result using the *t*-map data obtained the accuracy over 90% by performing the *N-*back task. This might be an indication that the memory-related neural degeneration is more apparent in the MCI patients when compared with the other mental functions. It will be interesting to apply another deep learning technique such as the recurrent neural network (RNN) (Sanchez et al., 2017; Li X. F. et al., 2018; Liu, 2018).

A number of different time intervals were evaluated in line with the statistical digital biomarkers in this study. As shown in Figures 5, 6 and Tables 3–8, the significant results (i.e., accuracy > 60% or *p*-value < 0.05) occurred randomly. It was difficult to conclude the best time interval for MCI detection. In addition, most of the studies (as listed in Table 1) prefer to conduct the statistical analysis using the entire task period between the groups of MCI and HC. However, as per the obtained results, the biomarkers were not consistent to make a satisfactory classification result. Therefore, the statistical analysis is not recommended to detect the early stage of AD. Therefore, as shown in Figure 9, the combined technique (deep learning and an imaging biomarker) shows a promising advantage for detecting the MCI patients from HC in the fNIRS field.

Since the present study accessed a relatively small number of MCI patients, no attempt was made to exclude patients based on other criteria. To substantiate the findings, research with a larger sample size would help ensuring that participants with secondary comorbidities can be excluded. In addition, a study with more participants will allow assessing separately, participants with different subtypes of MCI. In this study, we considered only the prefrontal lobes for our investigation, as PFC is widely (>90%) used for diagnosing MCI in the fNIRS area. Another issue for improvement can be found from the used headset. NIRSIT has a specific channel configuration for the PFC. It cannot be used over the entire brain. Meanwhile, several former studies claimed that MCI patients have a reduced activation in the hippocampus and PFC (Johnson et al., 2006; Dannhauser et al., 2008). A broader brain region than the PFC might give the better opportunity for examining more effective biomarkers. In the future, the whole brain with a hybrid technique including EEG and fNIRS (Khan et al., 2014, 2018; Hong et al., 2018b) with a greater number of subjects will be pursued hoping that more effective and reliable biomarkers for diagnosing the early stage of AD are disclosed.

## Conclusion

For the purpose of diagnosing MCI patients using fNIRS, we investigated three approaches (statistical analysis, LDA, CNN) in classifying the measured fNIRS signals. Fifteen digital biomarkers (i.e., 5 means and 7 slopes of ΔHbO/ΔHbR, peak time, skewness, kurtosis) in combination of LDA and two image biomarkers (*t*-map, correlation map) in combination with CNN were analyzed. It appears that the classical statistical analysis method is not reliable for clinical application, because the biomarkers (*p* < 0.05) that provided good LDA classification results (> 60%) were not consistent throughout the trials. However, the CNN classification result using the *t*-map input data provided the best classification accuracy (90.62%) between MCI and HC. Secondly, the local analyses in the PFC (left PFC, or middle PFC, or right PFC) provided better classification accuracies than examining the entire PFC. This leads to the conclusion that the task-related brain activity in the PFC may be localized per person, and the use of a few channels of fNIRS may be acceptable for MCI diagnosis. Finally, the *N*-back task presented a robust and accurate performance than the Stroop or VF tasks when the image biomarkers with CNN were analyzed.

## Data Availability

The datasets generated for this study are available on request to the corresponding author.

## Ethics Statement

This experiment was conducted in accordance with the latest Declaration of Helsinki upon the approval of the Pusan National University Institutional Review Board. All volunteers were given a detailed description of the experimental procedure prior to the beginning of the experiment, and they provided written consent agreeing to these experiments.

## Author Contributions

DY carried out the data processing and wrote the first draft of the manuscript. K-SH suggested the theoretical aspects of the current study, corrected the manuscript, and supervised the entire process leading to the manuscript generation. S-HY participated in collecting experimental data. C-SK has examined the data. All authors have approved the final manuscript.

## Conflict of Interest Statement

The authors declare that the research was conducted in the absence of any commercial or financial relationships that could be construed as a potential conflict of interest.
